# Applications of Solid-Phase Microextraction and Gas Chromatography/Mass Spectrometry (SPME-GC/MS) in the Study of Grape and Wine Volatile Compounds

**DOI:** 10.3390/molecules191221291

**Published:** 2014-12-18

**Authors:** Annarita Panighel, Riccardo Flamini

**Affiliations:** Consiglio per la Ricerca e la Sperimentazione in Agricoltura-Centro di Ricerca per la Viticoltura (CRA-VIT), Viale XXVIII aprile 26, Conegliano (TV) 31015, Italy; E-Mail: riccardo.flamini@entecra.it

**Keywords:** grape, wine, aroma, volatile compounds, SPME, GC/MS

## Abstract

Volatile compounds are responsible for the wine “bouquet”, which is perceived by sniffing the headspace of a glass, and of the aroma component (palate-aroma) of the overall flavor, which is perceived on drinking. Grape aroma compounds are transferred to the wine and undergo minimal alteration during fermentation (e.g., monoterpenes and methoxypyrazines); others are precursors of aroma compounds which form in winemaking and during wine aging (e.g., glycosidically-bound volatile compounds and C_13_-norisoprenoids). Headspace solid phase microextraction (HS-SPME) is a fast and simple technique which was developed for analysis of volatile compounds. This review describes some SPME methods coupled with gas chromatography/mass spectrometry (GC/MS) used to study the grape and wine volatiles.

## 1. Introduction

Wine aroma is formed by more than 800 volatile compounds and is characteristic of each product [1,2]. Often these compounds are present in very low concentration and are characterized by very low sensory thresholds (between ng/L and μg/L). Usually, the wine aroma profiling needs a sample preparation for isolation and concentration of the volatiles before performing gas chromatographic analysis. Several sample preparation methods for the analysis of grapes and wine were proposed: distillation [3,4], liquid–liquid extraction (LLE) [5,6], solid phase extraction (SPE) [7,8], dynamic headspace extraction [9], and headspace–solid phase microextraction (HS-SPME) [10,11,12,13].

SPME was developed in the 1990s by Pawliszyn and co-workers [14] and every year a thousand papers describing different aspects of this approach, and applications in different fields (chemical analysis, bioanalysis, food science, environmental science, and recently, pharmaceutical and medical sciences), are published [15]. This sample extraction technique was demonstrated to be rapid, simple, and reproducible, with no solvent use, and is suitable for the extraction and concentration of a high number of volatile and semi-volatile compounds from aqueous solutions [16,17]. Moreover, SPME needs a small sample volume and the coupling with gas chromatography and mass spectrometry (GC/MS) provides high sensitivity. For these reasons it has been used to study the volatile profile of many fruit varieties, vegetables, and beverages, including grapes and wine [18,19,20].

This paper reviews the main SPME-GC/MS applications developed to study the volatile and aroma compounds of grapes and wine.

### Grape and Wine Volatile Compounds

Since early 80’s a great number of studies of grape and wine volatiles have been performed and the main compounds identified are listed in Table 1. Principal in grape are monoterpenes, C_13_-norisoprenoids, benzene compounds, C_6_ aldehydes, and alcohols [21,22,23]. These compounds are present in berry skin and pulp in both free (volatile) and glycosidically-bound (non-volatile) form. Free volatile compounds directly contribute to grape and wine aroma while glycosides are flavorless compounds which can act as aroma precursors for enzymatic and acid hydrolysis occurring in winemaking and during wine storage [24].

In general, monoterpenes have floral and citrus notes; C_13_-norisoprenoids such as β-damascenone and β-ionone are characterized by “fruity-flowery”, “honey-like”, “sweet” and “violet” notes, respectively [25].

**Figure 1:**
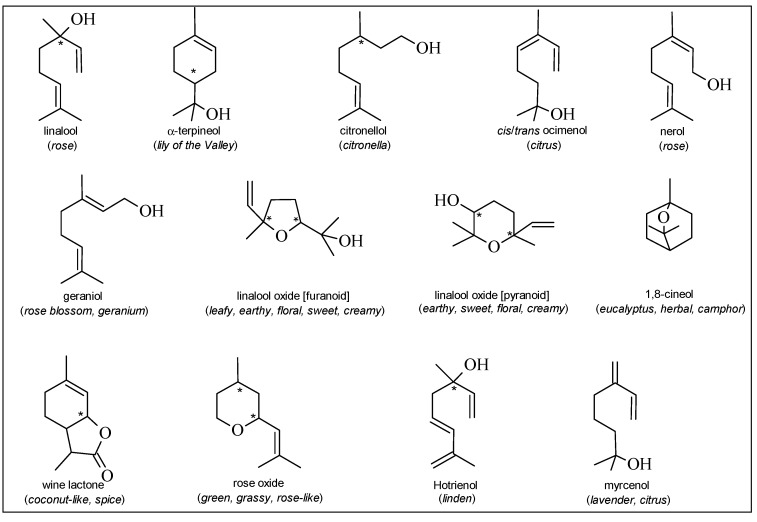
Principal monoterpenes identified in grapes. In brackets, the odor descriptor is reported [24].

**Figure 2:**
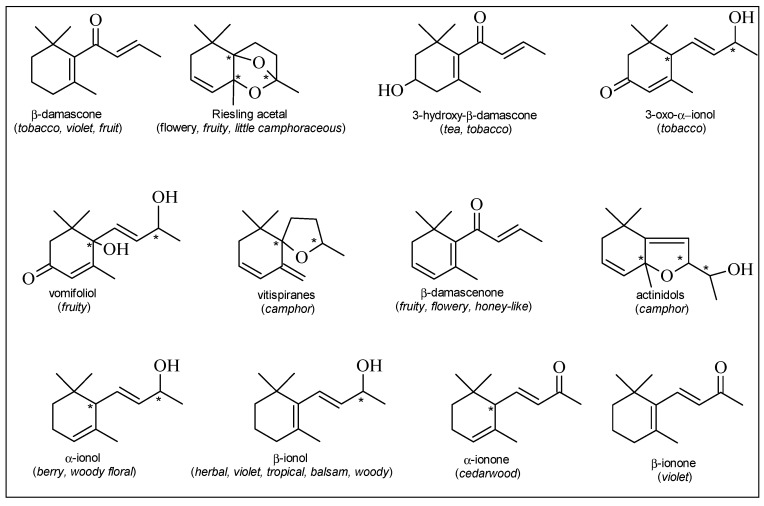
Principal C_13_-norisoprenoids identified in grapes. In brackets, the odor descriptor is reported [24].

**Figure 3:**
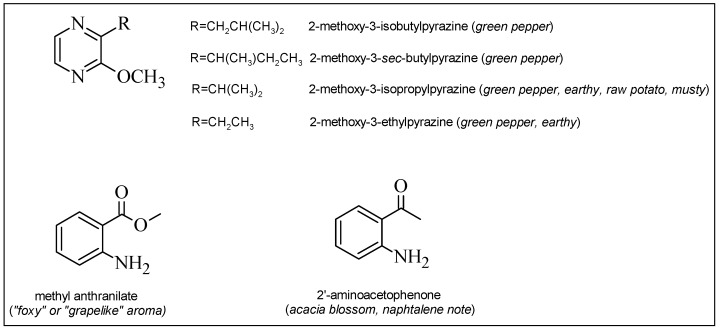
Structures of “foxy-smelling” compounds and alkyl methoxypyrazines in grapes. In brackets, the odor descriptor is reported [25].

Main grape and wine volatile benzenoids are aldehydes and alcohols, such as benzaldehyde, phenylacetaldehyde (hyacinth and rose-like odor [26]), vanillin, benzyl alcohol, and 2-phenylethanol [24], probably formed from L-phenylalanine via the shikimic pathway [27]. The structures of the main grape aroma compounds are shown in Figure 1, Figure 2 and Figure 3.

**Table 1:** Principal aroma compounds identified in grapes and wine [20,23,24,28,29].

**Terpenoids**	**Benzenoids**	**Sulfur compounds **
linalool	zingerone	methyl mercaptan
nerol	zingerol	ethyl mercaptan
geraniol	acetophenone	dimethyl sulfide
citronellol	vanillin	diethyl sulfide
α-terpineol	methyl salicylate	dimethyl disulfide
*cis*/*trans* ocimenol	eugenol	diethyl disulfide
*cis*/*trans* linalooloxide (furanic form)	*cis*/*trans* isoeugenol	methyl thioacetate
*cis*/*trans* linalooloxide (pyranic form)	2-phenylethanol	ethyl thioacetate
hydroxycitronellol	benzyl alcohol	2-mercaptoethanol
8-hydroxydihydrolinalool	acetovanillone	2-(methylthio)-1-ethanol
7-hydroxygeraniol	benzaldehyde	3-(methylthio)-1-propanol
7-hydroxynerol	4-hydroxybenzaldehyde	4-(methylthio)-1-butanol
*cis*/*trans* 8-hydroxylinalool	2,4-dimethylbenzaldehyde	2-furanmethanethiol
diendiol I	phenylacetaldehyde	benzothiazole
endiol	syringaldehyde	thiazole
diendiol II	coniferaldehyde	5-(2-hydroxyethyl)-4-methylthiazole
neroloxide	sinapaldehyde	4-methyl-4-mercaptopentan-2-one
2-*exo*-hydroxy-1,8-cineol	propriosyringone	3-mercaptohexanol acetate
1,8-cineol	propriovanillone	*cis*/*trans* 2-methylthiophan-3-ol
*cis*/*trans* 1,8-terpine	syringol	2-methyltetrahydrothiophen-3-one
*p*-menthenediol I	coniferyl alcohol	*cis*/*trans* 2-methyltetrahydrothiophen-3-ol
(*E*)-geranic acid	vanillic alcohol	3-mercaptohexan-1-ol
(*E*)-2,6-dimethyl -6-hydroxyocta-2,7-dienoic acid	sinapic alcohol	3-mercaptohexyl acetate
(*E*)- and (*Z*)-sobrerol	*o*-cymene	4-mercapto-4-methylpentan-2-ol
*cis*/*trans* rose oxide	*p*-cymene	3-mercapto-3-methylbutan-1-ol
lilac alcohols	guaiacol	
triol	4-ethylguaiacol	
hotrienol	4-vinylguaiacol	
myrcenol	4-ethylphenol	
limonene	4-vinylphenol	
β-phellandrene	methyl anthranilate	
β-ocimene	2'-aminoacetophenone	
wine lactone	
**Aliphatic alcohols**	**Acids**	**Sesquiterpenes**
1-butanol	isobutyric acid	rotundone
2-nonanol	isovaleric acid	farnesol
3-methyl-1-butanol	acetic acid	germacrene D
2-methyl-1-butanol	butyric acid	γ-cadinene
isobutanol	hexanoic acid	α-ylangene
1-pentanol	octanoic acid	α-farnesene
1-hexanol	decanoic acid	β-farnesene
1-octanol	hexadecanoic acid	nerolidol
(*E*)-3-hexen-1-ol	octadecanoic acid	
(*Z*)-3-hexen-1-ol		
4-methyl-3-penten-1-ol		
(*E*)-2-hexen-1-ol		
1-octen-3-ol		
2-ethyl-1-hexanol		
furfuryl alcohol		
6-methyl-5-hepten-2-ol		
**Carbonyl compounds**	**Esters**	**Norisoprenoids**
acetaldehyde	ethyl 2-methylpropanoate	TDN (1,1,6-trimethyl-1,2-dihydronaphthalene)
isobutyraldehyde	ethyl 2-methylbutanoate	β-damascone
2-methylbutanal	ethyl 3-methylbutanoate	β-damascenone
isovaleraldehyde	ethyl 2-hydroxypropanoate	vomifoliol
1-octen-3-one	ethyl 3-hydroxybutanoate	dihydrovomifoliol
(*E*)-2-heptenal	ethyl 4-hydroxybutanoate	3-hydroxy-β-damascone
methional	diethyl succinate	3-oxo-α-ionol
(*E*)-2-octenal	diethyl malate	3-hydroxy-7,8-dihydro-β-ionol
hexanal	ethyl butanoate	α-ionol
(*E*)-2-hexenal	ethyl hexanoate	β-ionol
(*Z*)-3-hexenal	ethyl octanoate	α-ionone
(*Z*)-2-nonenal	ethyl decanoate	β-ionone
furfural	ethyl benzoate	actinidols
5-methylfurfural	isoamyl octanoate	vitispiranes
1H-pyrrole-2-carboxyaldehyde	ethyl furoate	Riesling acetal
geranial	ethyl dihydrocinnamate	hydroxy-megastigmen-2-one
neral	ethyl cinnamate	hydroxy-megastigmen-3-one
acetoin	methyl vanillate	4-oxo-isophorone
diacetyl	ethyl vanillate	β-isophorone
glyoxal	ethyl acetate	4-oxo-2,3-dehydro-β-ionol
methylglyoxal	isobutyl acetate	β-cyclocitral
glycolaldehyde	isoamyl acetate	
hydroxypropandial	ethyl 2-phenylacetate	
2,4-nonadienal	hexyl acetate	
2,6-nonadienal		
**Lactones**	**Nitrogen compounds**	
γ-butyrolactone	3-isobutyl-2-methoxypyrazine	
γ-hexalactone	3-sec-butyl-2-methoxypyrazine	
γ-nonalactone	3-isopropyl-2-methoxypyrazine	
γ-decalactone	3-ethyl-2-methoxypyrazine	
*cis*/*trans* oak lactone		
sotolon		

Twenty-four aldehydes (mostly alkyls) were identified in wine; they can form during winemaking or be present in the grapes [28]. For example, glyoxal, methylglyoxal, hydroxypropandial and glycolaldehyde form by microorganisms such as *Saccharomyces cerevisiae* or *Leuconostoc oenos*, or can form in grapes as a consequence of *Botrytis cinerea* grape attack [30,31]. In general, C_6_ aldehydes (hexanal, (*E*)-2-hexenal, and (*Z*)-3-hexenal) and (*Z*)-2-nonenal are responsible for the green, herbaceous, and sometimes bitter aroma of wines [21]. They are mainly due to the enzymatic cleavage of oxidized linoleic and linolenic acid during grape crushing before fermentation [29].

Main wine volatiles are ethyl esters, acetates, and higher alcohols. Esters are produced by the yeasts during fermentation. Principal are ethyl esters characterized by fruity and floral notes, such as ethyl hexanoate, ethyl octanoate, ethyl decanoate, ethyl dodecanoate, isoamyl acetate, hexyl acetate, and 2-phenylethyl acetate [32,33]. Contents of hexanoic, octanoic, and decanoic acid in wine depend on the yeast strain, fermentation conditions and grape must composition [27]. Higher alcohols are formed by the yeast sugar metabolism (anabolic pathway) as well as *via* the catabolic or Ehrlich pathway of amino acids [27,34,35]. Rapp and Versini reported that a higher alcohols concentration below 300 mg/L is desirable for the aroma complexity of wine whereas a concentration exceeding 400 mg/L can have a detrimental effect [36].

Volatile phenols, such as 4-vinylphenol (aroma descriptors: spicy, pharmaceutical), 4-vinylguaiacol (smoke, phenolic), 4-ethylphenol (horse stable, medicinal), and 4-ethyl guaiacol (spice, phenolic), are produced during alcoholic fermentation by some microorganisms (*Brettanomyces* yeasts and bacteria) by decarboxylation of hydroxycinnamic acids present in must [32,33,37].

## 2. SPME-GC/MS Methods

### 2.1. Analysis of PFBOA-Derivatives

In general, carbonyl compounds contribute to the wine aroma even though they are present in low levels. GC/MS analysis of the *O*-pentafluorobenzyl (PFB) derivatives formed by reaction with *O*-(2,3,4,5,6-pentafluorobenzyl)-hydroxylamine (PFBOA) by recording in singular-ion-monitoring (SIM) the mass spectrum base peak signal at *m/z* 181 (characteristic of PFB-oximes) is a selective and sensitive method. On the other hand, derivatization increases the complexity of analyte peaks in the chromatogram due to formation of two oximes for each carbonyl group (isomers *E* and *Z*, except for formaldehyde) [38]. By this method several studies of carbonyl compounds in hydro-alcoholic matrices, such as wine, model wine solutions, and spirits, were performed [30,31,39,40,41,42,43,44,45,46,47].

Malolactic fermentation (MLF) is an important oenological process performed after the alcoholic fermentation for improving the organoleptic characteristics and the microbiological stability of wine [46]. The process is carried out by lactic acid bacteria: it can occur naturally or be induced by the inoculum of commercial bacteria strains. Conversion of L(−)-malic into L(+)-lactic acid decreases wine acidity [30]; usually, the inoculation of selected bacteria strains enables control over the process [48].

With MLF, together with the other fermentative compounds (esters, sulfur and nitrogen compounds, volatile phenols, and volatile fatty acids), also the carbonyl profile changes, increasing the aromatic complexity of wine [42,49].

HS-SPME and GC/MS analysis of PFBOA derivatives was performed to study the carbonyl compounds in Merlot wines after MLF [46]. In general, PFBOA derivatives are characterized by higher volatility, thermal stability, and affinity for the SPME fiber with respect to the corresponding carbonyl compounds [20]. SPME was performed by using a polydimethylsiloxane/divinylbenzene (PDMS/DVB) fiber; GC/MS analysis was performed by electron impact ionization (EI) and chemical ionization in both positive (PICI) and negative (NCI) mode [50]. NCI coupled to SPME provided lower detection limits for isobutyraldehyde, 2-methylbutanal, isovaleraldehyde, (*E*)-2-hexenal, 1-octen-3-one, (*E*)-2-heptenal, methional, (*E*)-2-octenal, phenylacetaldehyde, and (*E*)-2-nonenal [50]. PICI was used for determination of the principal carbonyl compounds in wine, such as acetaldehyde, diacetyl, and acetoin [46]. By using methane as reagent gas, abundant formation of acetaldehyde derivative [M+H]^+^ ion at *m/z* 240, diacetyl mono-derivative [M+H]^+^ ion at *m/z* 282, and *o*-chlorobenzaldehyde derivative [M+H]^+^ ion at *m/z* 336 (the internal standard), was observed. For acetoin abundant formation of [M+H-18]^+^ ion at *m/z* 266 was observed. These signals were used for the quantification of compounds. Experimental PICI conditions used are reported in Table 2 [46].

**Table 2:** Experimental conditions used for PFBOA derivatization and SPME-GC/MS analysis (ion trap and positive chemical ionization) of the main wine carbonyl compounds. Adapted from Flamini* et al.*, 2005 [46].

Sample volume	100 μL
Vial volume	4 mL
Derivatization conditions	200 μL IS *o*-chlorobenzaldehyde, 3.4 mg/L in ethanol/water solution; 1 mL of PFBOA 2 g/L aqueous solution, volume adjusted to 2 mL with water
SPME fiber	65-μm poly(ethylene glycol)/divinylbenzene (PEG/DVB)
Addition to the sample	50 mg NaCl
Sample heating	50 °C for 20 min under stirring
Extraction temperature and time	50 °C for 5 min
Desorption temperature and time	240 °C for 1 min
Fiber cleaning	250 °C for 5 min
GC column	HP-INNowax (30 m × 0.25 mm i.d; 0.25-μm film thickness)
Carrier gas	Helium, column headpressure 16 psi
Injector	T = 240 °C, sample volume 0.5 μL, splitless injection
Oven program	60 °C for 5 min, 3 °C/min to 210 °C, held 5 min
MS-IT conditions	PICI mode using methane as reagent gas (flow 1 mL/min), ion source at 200 °C, damping gas 0.3 mL/min, simultaneous SCAN (range *m/z* 40–660, 1.67 scan/s) and MS/MS
CID experiments	Collision gas He, excitation voltage 225 mV
Quantitative	Recorded signals at *m/z* 240 for acetaldehyde, *m/z* 282 for diacetyl, *m/z* 266 for acetoin, *m/z* 336 for *o*-chlorobenzaldehyde (I.S.)

### 2.2. Aroma Compounds and Wine Aging

Aging in wooden barrels is a process used to stabilize the color and to improve limpidity and the sensorial characteristics of wines. During aging many compounds are transferred from the wood to the wine: polyphenols, lactones, coumarins, polysaccharides, hydrocarbons and fatty acids, terpenes, C_13_-norisoprenoids, steroids, carotenoids, and furan compounds. Wood volatiles such as *cis*- and *trans*-β-methyl-γ-octalactones (oak lactones), syringaldehyde, vanillin, coniferaldehyde, sinapaldehyde and their alcohols, propiosyringone and propiovanillone, hydroxy-megastigmen-2-one and hydroxy-megastigmen-3-one [51], furfural, 5-methyl furfural, guaiacol, eugenol, 4-ethylphenol and 4-ethyl guaiacol [52,53], furfuryl alcohol, β-ionone, γ-noanalactone, and acetovanillone [54] confer the typical organoleptic characteristics of aged wines.

In making barrels for wine aging, oak (*Quercus sessilis*, *Q.* *petraea*, *Q.* *robur*,* Q.* *peduncolata*, *Q.* *alba*) is the wood more often used but other species, such as acacia (*Robinia pseudoacacia*), chestnut (*Castanea sativa*), cherry (*Prunus avium*), and mulberry (*Morus alba* and *Morus nigra*) are also being considered [55].

SPME-GC/MS was used to study the evolution of wine aroma during aging in 225-L barrels (barriques) made with these wood types. Experimental conditions used are reported in Table 3; main compounds identified are reported in Table 4 [55].

**Table 3:** SPME-GC/MS conditions used to study the evolution of volatile compounds of Raboso Piave wine during aging in five different types of wood barrels [55].

SPME fiber	65-μm carbowax/divinylbenzene (CAR/DVB)
Sample volume	10 mL
Vial volume	20 mL
Addition to the sample	3 g NaCl
Sample heating	70 °C for 10 min
Extraction temperature and time	70 °C for 30 min
Desorption temperature and time	230 °C fo 5 min
Fiber cleaning	10 min
GC column	HP-INNowax (30 m × 0.25 mm i.d; 0.25 μm film thickness)
Injection	Splitless
Oven program	40 °C for 5 min, 3 °C/min to 230 °C, held 10 min
MS conditions	ionization energy 70 eV, acquisition SIM mode

Wines aged in acacia, chestnut and oak wood showed higher contents of vanillin and eugenol and the acacia-aged sample showed an increase of 4-ethylguaiacol. Mulberry-aged wine had a significant decrease of 4-ethylguaiacol and increase of 4-ethylphenol; the wine aged in cherry barrel already showed high levels of 4-ethylguaiacol after three months of aging.

**Table 4:** Principal compounds studied in Raboso Piave wines aged nine months in 225 L barrels of acacia, cherry, chestnut, mulberry, and oak (nd: not detected; tr: trace (<0.01 ppm) [55]).

Barrel	Months of Aging	Compounds mg/L					
		Furfural	5-Methylfurfural	4-Ethylguaiacol	Eugenol	4-Ethylphenol	Vanillin
Acacia	3	0.02 ± 0.01	0.03 ± 0.01	2.24 ± 0.21	0.009 ± 0.001	0.67 ± 0.07	0.09 ± 0.03
	6	0.04 ± 0.01	0.03 ± 0.01	2.94 ± 0.14	0.015 ± 0.001	0.92 ± 0.08	0.16 ± 0.01
	9	0.03 ± 0.01	0.03 ± 0.01	3.25 ± 0.67	0.021 ± 0.005	1.29 ± 0.41	0.31 ± 0.07
Cherry	3	Nd	nd	3.01 ± 1.13	0.008 ± 0.004	1.00 ± 0.44	0.08 ± 0.04
	6	Tr	nd	3.13 ± 0.26	0.009 ± 0.001	1.04 ± 0.06	0.10 ± 0.01
	9	Nd	nd	2.79 ± 0.51	0.007 ± 0.001	0.86 ± 0.18	0.12 ± 0.03
Chestnut	3	0.04 ± 0.02	0.03 ± 0.01	2.53 ± 0.43	0.024 ± 0.004	0.84 ± 0.20	0.45 ± 0.06
	6	0.04 ± 0.01	0.02 ± 0.02	2.30 ± 0.12	0.035 ± 0.003	0.74 ± 0.08	0.60 ± 0.02
	9	0.07 ± 0.01	0.04 ± 0.01	1.84 ± 0.18	0.026 ± 0.002	0.64 ± 0.04	0.43 ± 0.03
Mulberry	3	Tr	nd	2.69 ± 0.75	0.004 ± 0.001	1.06 ± 0.26	0.09 ± 0.03
	6	Tr	nd	2.72 ± 0.44	0.006 ± 0.001	1.27 ± 0.26	0.08 ± 0.02
	9	Tr	tr	1.84 ± 0.20	0.006 ± 0.001	1.19 ± 0.07	0.08 ± 0.01
Oak	3	0.18 ± 0.08	0.14 ± 0.04	2.51 ± 0.14	0.009 ± 0.001	0.90 ± 0.07	0.27 ± 0.04
	6	0.56 ± 0.16	0.19 ± 0.05	2.08 ± 0.02	0.012 ± 0.003	0.75 ± 0.05	0.34 ± 0.08
	9	0.60 ± 0.06	0.32 ± 0.04	2.90 ± 0.75	0.018 ± 0.005	1.06 ± 0.36	0.36 ± 0.09

### 2.3. “Foxy Smelling Compounds” and 3-Alkyl-2-Methoxypyrazines in Grape Juice

2'-Aminoacetophenone (*o*-AAP) is the main compound identified as the cause of the aging note—the so-called “hybrid note”, “foxy-smelling” or “American character”—typical of *V.* *labruscana* grapes, even though it was also found in some *V. vinifera* wines such as Müller-Thurgau, Riesling, and Silvaner [56]. This note is variously described as “acacia blossom,” “naphthalene note,” “furniture polish,” “fusel alcohol,” and “damp cloth,” and causes a considerable number of wine rejections. The formation of *o*-AAP in grape is promoted by several factors, such as reduced nitrogen fertilization in combination with hot and dry summers, and the risk increases in wines made with grapes harvested early. The phytohormone indole-3-acetic acid (IAA) is the principal precursor of *o*-AAP through non-enzymatic processes [57,58]. Also, methyl anthranilate (MA) contributes to the typical foxy taint of wines made with American and wild vine grapes, although it was also found in some *V. vinifera* white wines in concentrations of up to 0.3 μg/L [59].

For analysis of *o*-AAP in wine a direct-immersion SPME method by using a DVB/CAR/PDMS fiber and GC/MS, was proposed [60]; instead, analysis of MA in grape juice was performed by using a PDMS fiber [61].

3-Alkyl-2-methoxypyrazines are present in the grape skin, pulp, and bunch stems. These compounds contribute to the aroma of wines by conferring vegetative, herbaceous, bell pepper, or earthy notes. They are characterized by very low sensory thresholds: for 3-isobutyl-2-methoxypyrazine (IBMP), 3-*sec*-butyl-2-methoxypyrazine (SBMP),and 3-isopropyl-2-methoxypyrazine (IPMP) are between 1 and 2 ng/L in water. The level of IBMP in wine may be 10 times the sensory threshold, whereas SBMP and IPMP are normally comparable to their sensory thresholds [62,63,64,65,66,67,68].

In general, the climate influences the biosynthesis of methoxypyrazines, and higher contents were found in grapes from cooler regions. For example, it was observed that 3-isobutyl-2-methoxypyrazine decreases dramatically during ripening. Methoxypyrazines may be also influenced by the light exposure: in general berries exposed to more sunlight have lower contents [67].

SPME-GC/MS of 3-alkyl-2-methoxypyrazines in grape juice and wine was performed by using DVB/CAR/PDMS, PDMS/DVB and CAR/PDMS fibers [69,70,71,72]. Also, a SPME-GC/MS and multiple mass spectrometry (MS/MS) method for simultaneous determination of *o*-AAP, MA, and the main four 3-alkyl-2-methoxypyrazines [ethylmethoxypyrazine (ETMP), IPMP, SBMP, and IBMP] in grape juice was proposed [73]. Quantitation of methoxypyrazines was performed on the signal area of MS/MS ions at *m/z* 119 (for ETMP), *m/z* 109 (IPMP), *m/z* 81 (IBMP), *m/z* 81 (SBMP) using 2-ethoxy-3-isopropylpyrazine as internal standard. For quantification of *o*-AAP and MA, as internal standard 2,4-dichloroaniline was used.

The optimized HS-SPME experimental conditions are described in Table 5 and GC/MS conditions in Table 6 [73].

**Table 5:** HS-SPME conditions used for simultaneous analysis of “foxy smelling compounds” (*o*-AAP and MA) and 3-alkyl-2-methoxypyrazines (ETMP, IPMP, IBMP, and SBMP) in grape juice [73].

SPME fiber	50/30 μm divinylbenzene/Carboxen^TM^/polydimethylsiloxane (DVB/CAR/PDMS)
Sample volume	10 mL
Vial volume	20 mL
Addition to the sample	3 g NaCl
Extraction temperature and time	50 °C for 30 min
Desorption temperature and time	250 °C fo 5 min
Fiber cleaning	10 min

**Table 6:** GC/MS conditions used for simultaneous analysis of *o*-AAP, MA and 3-alkyl-2-methoxypyrazines in grape juice [73]. MW: molecular weight.

GC column	HP-5ms: (5%-phenyl) methylpolysiloxane (30 m × 0.25mm i.d; 0.25-μm film thickness)			
Carrier gas	Helium at constant flow 1.2 mL/min			
Injector	250 °C			
Oven program	40 °C for 5 min, 5 °C/min to 230 °C, held 3 min			
MSD conditions	Ionization energy 70 eV, transfer line temperature 280 °C, ion source 250 °C, ion trap in MS/MS mode			
			IT-MS/MS	
			Precursor ion	MS/MS signal
Analyte	MW	GC retention time (min)	*m/z*	
3-ethyl-2-methoxypyrazine	138.17	15.10	138	119
3-isopropyl-2-methoxypyrazine	152.20	16.46	137	109
3-isobutyl-2-methoxypyrazine	166.22	18.90	124	81
3-sec-butyl-2-methoxypyrazine	166.22	19.14	138	81
2-ethoxy-3-isopropylpyrazine (IS)	166.22	18.45	166	123
methyl anthranilate	151.16	23.71	151	TIC
2'-aminoacetophenone	135.16	22.59	135	TIC
2,4-dichloroaniline (IS)	162.02	23.35	161	TIC

### 2.4. Volatile Phenols in Wine

4-Ethylphenol (4-EP) and 4-ethylguaiacol (4-EG) are associated with wine defects that can form during winemaking or, more commonly, wine aging. These compounds are characterized by sensorial characteristics described as “stable”, “animal” and “phenolic” and their presence is particularly detrimental for the product [74,75,76]. They are produced by winery contaminants, such as *Brettanomyces* and *Dekkera* yeasts, through processes of decarboxylation and reduction of ferulic and *p*-coumaric acids present in the grape [75]. Sensory thresholds of 4-EP and 4-EG in wine are 440 μg/L and 33 μg/L, respectively [77].

For their determination several sensitive SPME-GC/MS methods were proposed [20,78,79]. Martorell* et al*., proposed the use of two PDMS 100 μm fibers: for 4-EP the method had a limit of detection (LOD) and of quantification (LOQ) of 2 μg/L and 5 μg/L, respectively, for 4-EG 1 μg/L and 5 μg/L, respectively [80]. An optimized method for analysis of ethylphenols and vinylphenols in white and red wines was developed by the use of StableFlex Carbowax/DVB (CW/DVB 70 μm) and polyacrilate (PA 85 μm) fibers [79].

4-EG and 4-EP in wine were also analyzed by a multiple-headspace SPME method. By performing three consecutive extractions of the sample with a CW/DVB fiber, the possible matrix effects were minimized by providing a LOD of 0.06 μg/L for both 4-EG and 4-EP [81].

### 2.5. Higher Alcohols and Esters in Wine

By using a PDMS 100-μm fiber, effective methods for analysis of higher alcohols and aliphatic esters in wine were performed [82,83]. This coating fiber showed high affinity for non-polar compounds such as ethyl esters and acetates [84,85,86], while CW/DVB fiber is suitable for more polar compounds, such as 1-hexanol, hexen-1-ol, 1-octanol, and monoterpenols [84].

Antalick* et al*., developed a SPME and GC/MS-SIM method which provided simultaneous determination of 32 esters in wine in concentration between ng/L and mg/L [87]. Seven different fibers were tested: DVB/CAR/PDMS 50/30 μm, CAR/PDMS 85 μm, PDMS 100 μm, PDMS/DVB 65 μm, PA 85 μm, CW/DVB 70 μm, and polyethyleneglycol (PEG) 60 μm. PDMS was the most efficient in extracting the less polar and less volatile compounds; for more volatile esters the best coating was CAR/PDMS, and aromatic esters were better recovered by CW/DVB. In general, the PDMS fiber showed high efficiency for all compounds and provided LOQs between 0.4 ng/L and 4.0 μg/L.

Recent applications showed that the tri-phase fiber DVB/CAR/PDMS provides extraction of the highest number of wine volatiles, including ethyl esters (56% of the compounds identified), alcohols, and acids [2,88].

### 2.6. Wine Volatile Sulfur Compounds

Various sulfur compounds are present in wine, such as thiols, sulphides, thioesters, and heterocyclic compounds. Thiol and thio-type compounds are in general associated with flavor defects of wine and they are classified as “light” (boiling point < 90 °C) and “heavy” (b.p. > 90 °C) compounds [25,29,89,90,91]. Sulfur compounds can be formed through several enzymatic and non-enzymatic processes, such as yeast fermentation and chemical, photochemical, and thermal reactions occurring in winemaking and during wine storage [89,90]. Most prevailing are ethylmercaptan (EtSH; onion as aroma descriptor), dimethyl sulfide (DMS; grassy/truffle-like note), 2-furanmethanethiol (FFT; roasted coffee), diethyl sulfide (DES; cooked vegetables, onion, garlic), dimethyl disulfide (DMDS; cooked gabbage, intense onion), diethyl disulfide (DEDS; garlic, burnt rubber), methyl thioacetate (MTA), ethyl thioacetate (ETA), 2-mercaptoethanol (ME; burnt rubber), 2-(methylthio)-1-ethanol (MTE; cauliflower), 3-(methylthio)-1-propanol (MTP; sweet, potato), 4-(methylthio)-1-butanol (MTB; earthy-like scent), benzothiazole (BT; rubber), and 5-(2-hydroxyethyl)-4-methylthiazole (HMT) [29,91]. Also, 2-methyl-3-furanthiol (MF; cooked meat), a very odoriferous compound with an odor threshold of 0.4–1.0 ppt, was found [92].

A SPME method for analysis of 13 volatile sulfur compounds in wine (*i.e.*, DMS, EtSH, DES, MTA, ETA, ME, DMDS, DEDS, BT, HMT, MTB, MTP, and MTE) with b.p. ranging from 35 °C to 231 °C by using a CAR/PDMS/DVB 50:30 μm 2 cm length fiber, was developed [90]. By addition of MgSO_4_ (1.0 M) to increase ionic strength of solution and performing the extraction at 35 °C, the method showed a high sensitivity for all the analytes.

3-mercaptohexan-1-ol (3-MH; passion fruit, grapefruit), 3-mercaptohexyl acetate (3-MHA; Riesling type note, passion fruit, box tree), and 4-methyl-4-mercaptopentan-2-one (4–MP; blackcurrant or box tree note) are tropical fruit scenting volatiles present in wines at ng/L level [29]. A SPME-GC/MS method for analysis of these compounds by using a CAR/PDMS/DVB fiber was proposed [93]. HS-SPME conditions were optimized by performing the extraction of wine adjusted to pH 7 at 40 °C for 40 min [93].

A method for analysis of thiols in wine by synthesis of the pentafluorobenzyl derivatives was also proposed. Derivatization was performed directly on the PDMS/DVB fiber (65 μm), LODs achieved were 0.05 ng/L, 0.03 ng/L, 0.11 ng/L, 0.5 ng/L, and 0.8 ng/L for FFT, 3-MHA, MF, 4-MP, and 3-MH, respectively [94].

## 3. Conclusions

Grape aroma is composed of a hundred compounds and wine’s volatile profile also includes a number of fermentative compounds. SPME coupled to GC/MS showed to be effective for studying several classes of these analytes without solvent use. It often resulted in a high-sensitive technique for quantitative analysis of compounds for which the standard is available with high reproducibility. Moreover, the use of a multiphase fiber coupled to MS and multivariate data analysis allows sampling automation and statistical treatment of fragment abundances for the identification of compounds [95,96].

On the other hand, different to most of the sample preparation methods performed by liquid-liquid extraction and SPE, the selectivity of SPME fiber often changes dramatically for the different analytes. As a consequence, it is rarely possible to perform the semi-quantitative profiling of the sample on the internal standard signal, which is particularly useful in the characterization of grape varieties and the monitoring of the winemaking processes.

Logically, by increasing the standards commercially available new SPME-GC/MS applications are developed, and methods for the profiling of specific classes of grape aroma compounds, such as terpenols and norisoprenoids, could be particularly useful.
